# C1q/Tumor Necrosis Factor-Related Protein-3 Attenuates Brain Injury after Intracerebral Hemorrhage via AMPK-Dependent Pathway in Rat

**DOI:** 10.3389/fncel.2016.00237

**Published:** 2016-10-19

**Authors:** Shaohua Wang, Yang Zhou, Bo Yang, Lingyu Li, Shanshan Yu, Yanlin Chen, Jin Zhu, Yong Zhao

**Affiliations:** ^1^Department of Pathology, Chongqing Medical UniversityChongqing, China; ^2^Key Laboratory of Neurobiology, Chongqing Medical UniversityChongqing, China

**Keywords:** CTRP3, intracerebral hemorrhage, brain edema, blood-brain barrier disruption, neuroprotection, angiogenesis

## Abstract

C1q/tumor necrosis factor (TNF)-related protein-3 (CTRP3) is a recently discovered adiponectin paralog with established metabolic regulatory properties. However, the role of CTRP3 in intracerebral hemorrhage (ICH) is still mostly unresolved. The aim of the present report was to explore the possible neuroprotective effect of CTRP3 in an ICH rat model and to elucidate the fundamental mechanisms. ICH was induced in rats by intracerebral infusion of autologous arterial blood. The effects of exogenous CTRP3 (recombinant or lentivirus CTRP3) on brain injury were explored on day 7. Treatment with CTRP3 reduced brain edema, protected against disruption of the blood-brain barrier (BBB), improved neurological functions and promoted angiogenesis. Furthermore, CTRP3 greatly intensified phosphorylation of AMP-activated protein kinase (AMPK) in addition to expression of hypoxia inducing factor-1α (HIF-1α) and vascular endothelial growth factor (VEGF). Finally, the protective effects of CTRP3 could be blocked by either AMPK or VEGF inhibitors. Our findings give the first evidence that CTRP3 is a new proangiogenic and neuroprotective adipokine, which may exert its protective effects at least partly through an AMPK/HIF-1α/ VEGF-dependent pathway, and suggest that CTRP3 may provide a new therapeutic strategy for ICH.

## Introduction

Intracerebral hemorrhage (ICH) is responsible for about 10–15% of stroke cases. Nevertheless, it is the most alarming type due to its large fatality rate and poor functional outcome (Qureshi et al., 2009; Manaenko et al., 2013). ICH results from rupture of blood vessels in the brain. The quick release of blood into the parenchyma leads to substantial mechanical damage that may only be somewhat ameliorated by limiting hematoma volume (Mayer et al., 2008). Meanwhile, secondary damage ensues due to toxic effects of released blood components, such as thrombin, as well as erythrocyte rupture (for example, iron-catalyzed free radical reactions). These events, including disruption of the blood-brain barrier (BBB) as well as growth of edema and inflammation, are currently therapeutic targets for ICH (Fingas et al., 2009; Keep et al., 2012).

Therapies such as angiogenesis have been suggested for ICH. Growing evidence suggests that after ICH, angiogenesis is upregulated in damaged brain tissue of the peri-hematoma area, leading to compensatory cerebral vascular network remodeling (Teng et al., 2008; Lei et al., 2015). Past reports showed that many angiogenic aspects could ease ischemic brain injury, elevate focal blood flow and enhance neurological results, which indicate that recently formed microvessels are indeed functional (Hao et al., 2011; Shen et al., 2013). Blood vessels are a significant scaffolding factor that assist with the migration of neurons to damaged brain areas and supply trophic material to new neurons (Kojima et al., 2010; Lei et al., 2013). The molecules that encourage neurogenesis and angiogenesis after brain injuries (e.g., ICH) are still unidentified.

Recently, a highly conserved family of adiponectin paralogs, C1q/tumor necrosis factor(TNF)-related proteins (CTRPs), was identified. Each of the 15 known members (CTRP1–CTRP15) is made up of four separate domains including an N-terminal signal peptide, a short variable domain, a collagen-like domain, and a C-terminal C1q-like globular domain (Ahima et al., 2006). Both CTRPs and adiponectin are a part of the C1q/TNF protein superfamily, which proceeds to increase in size as more C1q domain proteins are identified (Yi et al., 2012). The CTRP family members exhibit broadly diverse physiological functions, including regulation of metabolism, protection against endothelial dysfunction and angiogenesis.

CTRP3 is ubiquitously expressed in adipocytes, cartilagocytes, monocytes, fibroblasts, placenta, osteosarcoma, chondroblastoma, giant cell tumor, colon, small intestine, pancreas, kidney, thymus, ovary and in brain (Schaffler and Buechler, 2012). Most importantly, CTRP3 is the only one whose biological functions have been identified (Peterson et al., 2010). It was found that CTRP3 can encourage *in vitro* endothelial cell proliferation and migration (Akiyama et al., 2007). But, the role of CTRP3 in promoting angiogenesis in ICH-induced brain injury is not yet known. Further, whether or not CTRP3, an important member of the most recently discovered adipokine family, works as a mediator or inhibitor of ICH has not been studied previously. Therefore, the goals of this research were: (1) to investigate the effects of exogenous CTRP3 in an ICH rat model; (2) to determine whether CTRP3 administration promotes angiogenesis after ICH; and (3) to elucidate the role of CTRP3 in pathogenesis of ICH.

## Materials and Methods

### Experimental Animals

All animal studies were given approval by the Chongqing Medical University Biomedical Ethics Committee. All experimental procedures were done in accordance with the National Institutes of Health Guide for the Care and Use of Laboratory Animals. All efforts were made to minimize the number of animals used and their suffering. A total of 115 adult male Sprague-Dawley rats (60–80 d old, 240–300 g) were used for the *in vivo* study.

### Establishment of Intracerebral Hemorrhage Model

ICH was induced by an intrastriatal blood infusion method as described previously (Ni et al., 2015). Briefly, rats were deeply anesthetized with chloral hydrate (350 mg/kg, intraperitoneal injection) and placed in a stereotaxic frame (Kopf Instruments, Tujunga, CA, USA). After removing the hair and cleaning the scalp, the skin was incised. A burr hole was drilled 0.2 mm anterior and 3.0 mm lateral right of bregma. Whole blood (50 μL), which was drawn from the femoral artery, was infused manually over 10 min via a 26 G needle inserted into the striatum at a depth of 5.8 mm below the surface of the skull. After 10 min, the needle was steadily taken out for 5 min followed by the sealing of the burr hole with a sterilized medical bone wax. The wound was cleaned, and the scalp was sutured. The animals were given time to heal in their cages. During the recovery period, the animals had unlimited access to nourishment.

### *In vivo* Experiments

Rats were given free access to food and water in an optimal environment preceding the operation. Three *in vivo* experiments were performed as described below.

#### Experiment 1

Adult rats were split at random into the following four groups: sham-operated (sham) group, ICH group, ICH + vehicle group and ICH + recombinant CTRP3 (rCTRP3, Chimerigen, USA) group. rCTRP3 was injected intracerebroventricularly (80 μg/kg) 30 min after ICH and then three times per week until the animals were killed. Neurological deficits (assessed by a modified Garcia test, beam walking test and wire hanging test), hematoma volume, BBB integrity and brain edema were measured 7 days after ICH, and samples for western blot, qRT-PCR and immunohistochemistry were collected.

#### Experiment 2

Adult rats were split at random into the following four groups: sham-operated (sham) group, ICH group, ICH + null vector control (Lenti.null) group, ICH + lentivirus overexpression of CTRP3 (Lenti.CTRP3) group. Fourteen days after Lenti.CTRP3 intracerebroventricular injection, the rats underwent ICH. Neurological deficits, hematoma volume, BBB integrity and brain edema were measured 7 days after ICH, and samples for western blot, qRT-PCR and immunohistochemistry were collected.

#### Experiment 3

Adult rats were split at random into the following four groups: ICH group, ICH + rCTRP3 group, ICH + rCTRP3 + compound C (Com.C) group (AMP-activated protein kinase (AMPK) axis inhibitor, 20 μg/kg, intracerebroventricular injection, 3 times per week), and ICH + rCTRP3 + CBO-P11 (CBO) group (vascular endothelial growth factor (VEGF) inhibitor 40 μg/kg, intracerebroventricular injection, 3 times per week). Neurological deficits and BBB integrity were measured 7 days after ICH, and samples for western blot and immunohistochemistry were collected.

### Lentivirus-CTRP3 Gene Transfer in the Rat Brain

Adult rats were anesthetized with chloral hydrate (350 mg/kg intraperitoneal injection) and then placed in a Kopf stereotactic frame. A burr hole was bored in the pericranium 0.9 mm lateral to the sagittal suture and 1.9 mm posterior to the coronal suture. A 10 μL microinjection pump (WPI Inc., Sarasota, FL, USA) was stereotactically inserted 3.5 mm deeper into the cortex. A 5 μL viral suspension consisting of 1 × 10^9^ genomic copies of the lentivirus-CTRP3 (Lenti-CTRP3) gene, which was injected ipsilaterally into the right lateral cerebral ventricle at a rate of 0.2 μL/min. The needle was taken out after 15 min of injection. The animals then were allowed to heal and brought back to their cages.

### Behavioral Assessment

Behavioral functions were measured using a modified Garcia test, beam walking test, and wire hanging test 7 days after ICH in a blind fashion (Chen et al., 2013). In the modified Garcia test, rats were given a score of 0–18. The scoring system consisted of six tests, with possible scores of 0–3 (0 = worst; 3 = best). The minimum score was 0 and the maximum was 18. Beam walking and wire hanging tests utilized bridges (550 cm wire or 590 cm beam) between two platforms on which the rats were placed in the center. Rats were assessed according to six criteria that described if the animal could reach the platform and use its limbs in a symmetrical manner and were then given a score of 0–5 (normal). The average of three trials per test for each animal was calculated.

### BBB Permeability

Seven days after ICH, rats were intravenously injected with 2% Evans blue dye (4 mL/kg; Sigma-Aldrich, St. Louis, MO, USA). Three hours later, the amount of extravasated Evans blue dye in the hemorrhagic brain hemispheres was evaluated by spectrophotometry (Thermo Scientific, MA, USA) at 620 nm (Cai et al., 2015).

### Brain Water Content

Seven days after ICH, the cerebral hemisphere was cut into 4-mm thick blocks around the needle track. Brain tissues were immediately weighed using an analytical balance and heated at 100°C for 24 h to obtain the dry weight. The water content was calculated using the following formula: (wet weight– dry weight)/wet weight × 100%.

### Hematoma Volume

Hematoma volume was evaluated using a spectrophotometric hemoglobin assay 7 days after the ICH operation (Ma et al., 2014).

### Western Blot Analysis

Total protein was extracted from the peri-hematoma area of the rat striatum using cell lysis buffer supplemented with proteinase and phosphatase inhibitors. Cell lysates were split by 10% SDS-PAGE and transferred to polyvinylidene fluoride membranes. Then, the membranes were blocked in 5% non-fat milk TBST buffer for 1.5 h at room temperature. The membranes were incubated in primary antibody overnight at 4°C and in secondary antibody for 1 h at room temperature. Dilutions for primary antibodies were the following: anti-vascular endothelial growth factor (VEGFA; 1:1000, Abcam, Cambridge, MA, USA), anti-hypoxia inducing factor-1α (HIF-1α; 1:500, Abcam, Cambridge, MA, USA), and anti-AMPK (phospho-thr172; 1:500, Bioworld, Dublin, OH, USA). The secondary antibody was diluted 1:5000 (Sangon Biotech, Shanghai, Co., Ltd.). The density of bands was detected using an imaging densitometer (Bio-Rad, Foster City, CA, USA), and the gray value of bands was quantified using Quantity One 1-D analysis software (Bio-Rad).

#### qRT-PCR

Total RNA was removed with RNAiso Plus (TaKaRa Biotechnology, Dalian, China) using the manufacturer’s instructions. Reverse transcription was done with a cDNA synthesis kit (TaKaRa Biotechnology). Real-time PCR reactions were performed with TaKaRa SYBR Premix Ex Taq II (TliRnaseH Plus, TaKaRa Biotechnology) on a PCR amplifier (CFX-96 Content Real-time System). Primers (Sangon Biotech) are recorded in Table 1.

**Table 1:** **Primers used in qRT-PCR**.

Gene product	Forward primer	Reverse primer	Fragment size (bp)
CTRP3	5′-ATGGAGGTGAGCAGAAGAGC-3′	5′CACAGTCCCCGTTTTAGCAT-3′	126
HIF-1α	5′-CTCCCTTTTTCAAGCAGCAG-3′	5′GCTCCATTCCATCCTGTTCA-3′	125
VEGFA	5′-CGTCCTGTGTGCCCCTAAT-3′	5′TGGCTTTGGTGAGGTTTGAT-3′	121
β-actin	5′-TGTTTGAGACCTTCAACACC-3′	5′-CGCTCATTGCCGATAGTGAT-3′	207

#### Immunohistochemistry

Rats were killed 7 days after ICH induction by intraperitoneal injection of chloral hydrate. Immunohistochemistry was performed as previously described (Lei et al., 2013; Lapi et al., 2015). The primary antibody used was rabbit anti-rat CD31 (1:100, Abcam, USA). Total vessel densities were computed by counting 4 areas in 3 sections through the stroke region for each animal. Sections were stained with antibody against new vessel marker CD31; positive staining appeared brown. Standard quantitation was done as percent CD31-positive in the region bordering the hematoma.

#### Statistical Analysis

All data are given as mean ± S.E.M. One-way analysis of variance (ANOVA) followed by Student’s *t* test was utilized to collate outcomes among all groups. The SPSS 17.0 software package was utilized to do all statistics. *P* < 0.05 was considered statistically significant.

## Results

### CTRP3 Reduced Brain Edema and Improved Functional Outcomes after ICH

**Figure 1:**
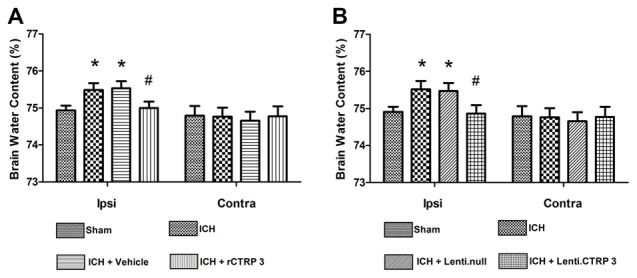
**Effects of CTRP3 treatment on brain water content 7 days after ICH. (A)** Brain water content in ipsilateral (Ipsi) and contralateral (Contra) hemispheres 7 days after ICH in rats treated with rCTRP3. **(B)** Brain water content in ipsilateral (Ipsi) and contralateral (Contra) hemispheres 7 days after ICH in rats treated with Lenti-CTRP3. Values are mean ± SEM. *n* = 8 per group. *p < 0.05 vs. sham; ^#^*p* < 0.05 vs. vehicle or Lenti.null. CTRP3, C1q/tumor necrosis factor-related protein-3; rCTRP3, recombinant CTRP3; Lenti.CTRP3, Lentivirus overexpression of CTRP3; ICH, intracerebral hemorrhage.

**Figure 2:**
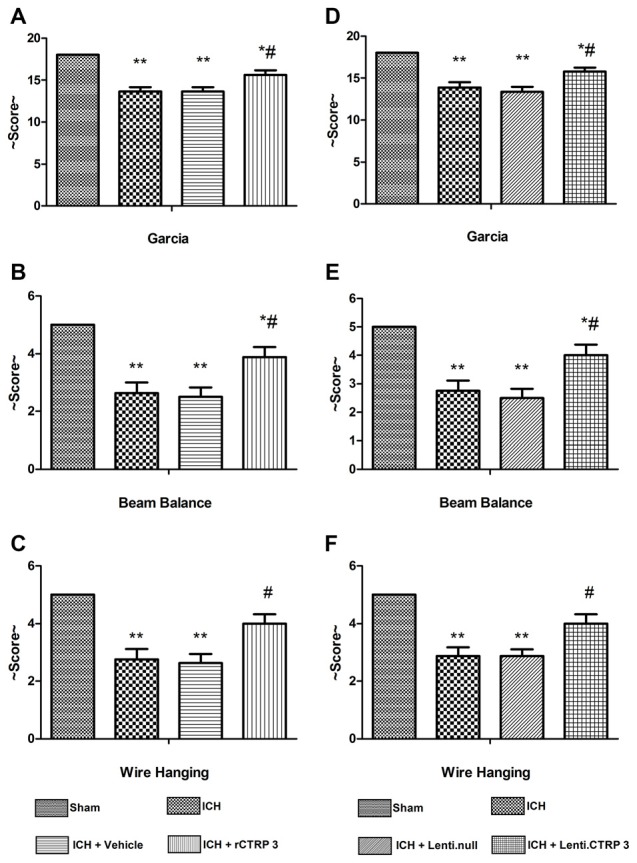
**Effects of CTRP3 treatment on neurological functions 7 days after ICH.** All animals after ICH showed significant neurological deficits based on performance on the modified Garcia **(A)**, beam balance **(B)** and wire hanging **(C)** tests. Rats treated with rCTRP3 showed reduced neurological deficits in all three tests. Treatment with Lenti-CTRP3 starting 2 weeks before induction of ICH showed a tendency to ameliorate neurological deficits **(D–F)**. Values are mean ± SEM. *n* = 9 per group. *p < 0.05 vs. sham; **p < 0.01 vs. sham; ^#^*p* < 0.05 vs. vehicle or Lenti.null. CTRP3, C1q/tumor necrosis factor-related protein-3; rCTRP3, recombinant CTRP3; Lenti-CTRP3, Lentivirus overexpression of CTRP3; ICH, intracerebral hemorrhage.

Quantification of brain water content showed that ICH rats had significantly greater edema in the ipsilateral hemisphere than sham-operated rats (Figure 1A). Brain edema in the ipsilateral hemisphere was significantly less in rCTRP3-treated rats than in vehicle-treated rats (Figure 1A). In the contralateral hemisphere, rCTRP3 failed to affect brain water content (Figure 1A). Subsequently, we tested functional outcomes using a battery of behavioral tests in rats treated with rCTRP3 or vehicle. Neurological deficits were significantly more severe in all ICH vs. sham animals 7 days after ICH as tested by the modified Garcia test (Figure 2A), wire hanging test (Figure 2B), and beam balance test (Figure 2C). A statistically significant advancement was seen in all three neurobehavioral tests after rCTRP3 treatment.

Similarly, Lenti.CTRP3 treatment decreased brain water content in ICH rats (Figure 1B). Statistically significant neurological shortfalls were observed in all ICH vs. sham animals 7 days after ICH. Treatment with Lenti-CTRP3 tended to ameliorate neurological deficits 7 days after ICH (Figures 2D–F). Both the rCTRP3 and Lenti.CTRP3 treatments were effective.

### CTRP3 Reduced Disruption of the BBB

**Figure 3:**
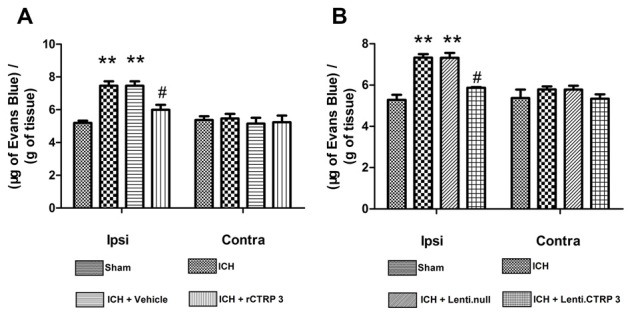
**Effects of CTRP3 treatment on ICH-induced blood-brain barrier (BBB) damage 7 days after ICH. (A)** Quantification of Evans blue dye extravasation (blue staining) in the ipsilateral (Ipsi) and contralateral (Contra) hemispheres 7 days after ICH in rats treated with rCTRP3. **(B)** Quantification of Evans blue dye extravasation (blue staining) in ipsilateral (Ipsi) and contralateral (Contra) hemispheres 7 days after ICH in rats treated with Lenti-CTRP3. Values are mean ± SEM. *n* = 6 per group. **p < 0.01 vs. sham; ^#^*p* < 0.05 vs. vehicle or Lenti.null. CTRP3, C1q/tumor necrosis factor-related protein-3; rCTRP3, recombinant CTRP3; Lenti-CTRP3, Lentivirus overexpression of CTRP3; ICH, intracerebral hemorrhage.

We measured BBB permeability by Evans blue extravasation in ICH rats. Significant accumulation of Evans Blue stain was seen in the ipsilateral hemisphere of ICH in comparison to sham animals (Figure 3A). Treatment with rCTRP3 significantly reduced the quantity of stain in the ipsilateral hemisphere in comparison to the vehicle 7 days after ICH (Figure 3A). Rats were similarly protected when treatment with Lenti-CTRP3 also caused a significant reduction in BBB permeability (Figure 3B).

### Effect of CTRP3 on the Hematoma Volume-Hemoglobin Assay

Neither recombinant nor lentivirus CTRP3 treatment had any obvious effect on hemorrhage volume at 7 days (data not shown).

### CTRP3 Promotes Angiogenesis and Activates the AMPK/HIF-1α/VEGF Axis in the ICH Brain

**Figure 4:**
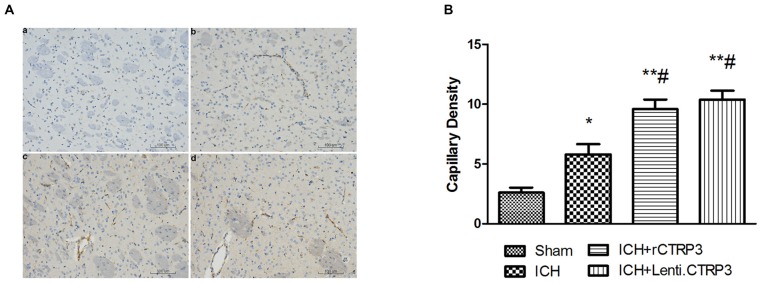
**CTRP3 encourages angiogenesis in the hematoma border zone 7 days after ICH. (A)** Capillary density computed by immunohistochemical staining for CD31 (brown) in the perifocal region in ICH **(b)**, rCTRP3 **(c)** and Lenti-CTRP3 **(d)** 7 days after ICH. A control brain section demonstrates a normal distribution of microvessels in the same region **(a)**. Bar = 100 μm. **(B)** Bar graph showing the number of brown stained capillaries 7 days after ICH. Values are mean ± SEM. *n* = 5 per group. *p < 0.05 vs. sham; **p < 0.01 vs. sham; ^#^*p* < 0.05 vs. ICH. CTRP3, C1q/tumor necrosis factor-related protein-3; rCTRP3, recombinant CTRP3; Lenti-CTRP3, Lentivirus overexpression of CTRP3; ICH, intracerebral hemorrhage; CD31, platelet endothelial cell adhesion molecule-1.

Angiogenesis is a vital part in beginning brain recovery after ICH. Having shown CTRP3 as an innovative adipokine involved in recovery following ICH, we assessed angiogenic effects of CTRP3. As illustrated in Figures 4A,B, CTRP3 treatment significantly enhanced the amount of CD31-positive capillary vessels in the zone bordering the hematoma 7 days after ICH, pointing to the observation that CTRP3 encourages vessel formation following ICH.

**Figure 5:**
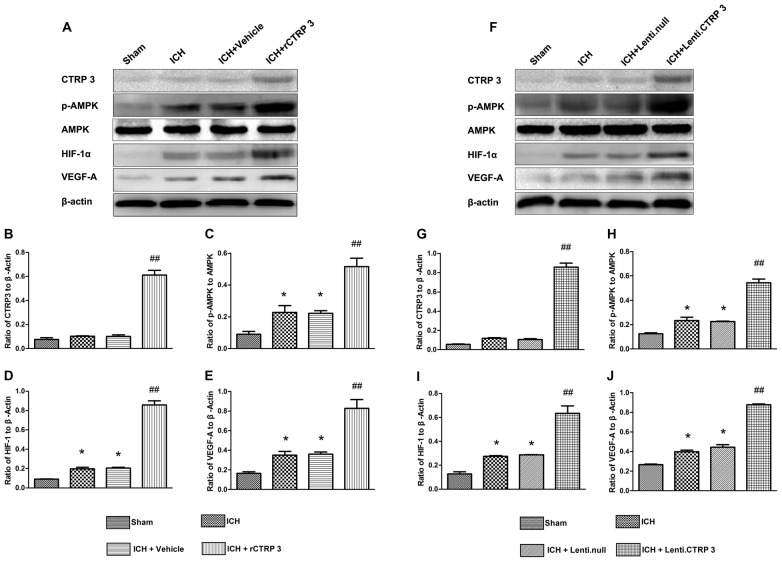
**CTRP3 upregulates pAMPK/HIF-1α/vascular endothelial growth factor (VEGF) axis protein expression in the hematoma border zone 7 days after ICH. (A)** Western blot analysis of the effect of rCTRP3 on CTRP3, phosphorylated (p) AMP-activated protein kinase (AMPK; Thr172)/AMPK, HIF-1α and VEGF-A. **(B–E)** Representative ratios of CTRP3, p-AMPK, HIF-1α and VEGF-A protein expression. **(F)** Western blot analysis of the effect of Lenti-CTRP3 on CTRP3, phosphorylated (p) AMPK (Thr172)/AMPK, HIF-1α and VEGF-A. **(G–J)** Representative ratios of CTRP3, p-AMPK, HIF-1α and VEGF-A protein expression. Values are mean ± SEM. *n* = 4 per group. *p < 0.05 vs. sham; ^##^*p* < 0.01 vs. vehicle or Lenti.null. CTRP3, C1q/tumor necrosis factor-related protein-3; rCTRP3, recombinant CTRP3; Lenti-CTRP3, Lentivirus overexpression of CTRP3; ICH, intracerebral hemorrhage.

**Figure 6:**
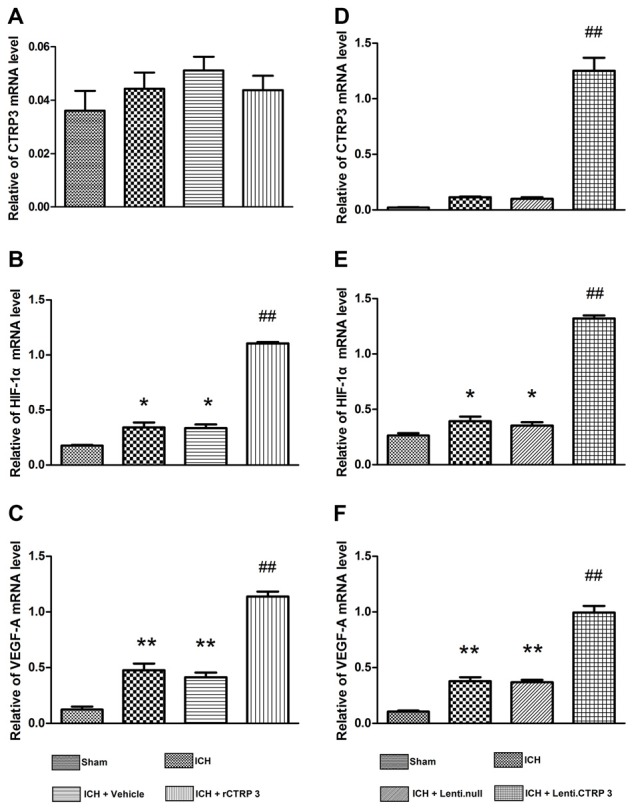
**Quantitative RT-PCR analysis of HIF-1α and VEGF-A mRNA levels in the hematoma border zone 7 days after ICH. (A–C)** Quantitative RT-PCR analysis of the effect of rCTRP3 on CTRP3, HIF-1α and VEGF-A mRNA levels. **(D–F)** Quantitative RT-PCR analysis of the effect of rCTRP3 on CTRP3, HIF-1α and VEGF-A mRNA levels. Values are mean ± SEM. *n* = 4–6 per group. *p < 0.05 vs. sham; **p < 0.01 vs. sham; ^##^*p* < 0.01 vs. vehicle or Lenti.null. CTRP3, C1q/tumor necrosis factor-related protein-3; rCTRP3, recombinant CTRP3; Lenti.CTRP3, Lentivirus overexpression of CTRP3; ICH, intracerebral hemorrhage.

To further figure out the signaling pathways in charge of increased angiogenesis after CTRP3 treatment, many critical mediators and cytokines required for angiogenesis were tested. rCTRP3 significantly enhanced AMPK phosphorylation and increased HIF-1α and VEGF expression (Figures 5A–E). Similar changes in mediator and cytokine expression were found when animals were treated with lentivirus CTRP3 (Figures 5F–J). The results from qRT-PCR were in agreement with those from Western blots (Figure 6). These outcomes imply that CTRP3 promotes angiogenesis after ICH possibly through the AMPK/HIF-1α/VEGF axis.

### Inhibiting AMKP or VEGF Activation Attenuated Effects of Recombinant CTRP3

**Figure 7:**
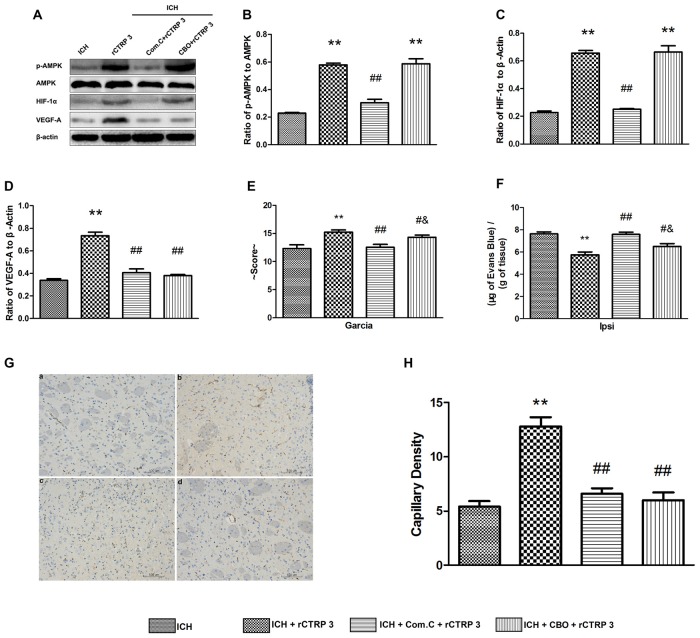
**CTRP3 promotes angiogenesis via an AMPK-dependent signaling mechanism. (A)** Western blot analysis of the effects of COM.C and CBO on rCTRP3-induced p-AMPK, HIF-1α and VEGF-A expression. **(B–D)** Representative ratios of p-AMPK, HIF-1α and VEGF-A protein expression. **(E)** Modified Garcia test analysis of the effects of COM.C and CBO on neurological function improvement induced by rCTRP3. **(F)** Evans blue dye extravasation analysis of the effects of COM.C and CBO on ICH-induced BBB damage reduced by rCTRP3. **(G)** Immunohistochemical staining analysis of the effects of COM.C and CBO on angiogenesis induced by rCTRP3. Capillary density measured by immunohistochemical staining for CD31 (brown) in the perifocal region in ICH **(a)**, rCTRP3 **(b)**, COM.C **(c)** and CBO **(d)** 7 days after ICH. **(H)** Bar graph showing the number of brown stained capillaries. Values are mean ± SEM. *n* = 4–6 per group. **p < 0.01 vs. ICH; ^#^*p* < 0.05 vs. rCTRP3; ^##^*p* < 0.01 vs. rCTRP3; ^&^*p* < 0.05 vs. COM.C. CTRP3, C1q/tumor necrosis factor-related protein-3; rCTRP3, recombinant CTRP3; Lenti-CTRP3, Lentivirus overexpression of CTRP3; ICH, intracerebral hemorrhage; COM.C, compound C; CBO, CBO-P11.

To determine whether the AMPK/HIF-1α/VEGF signaling pathway is accountable for CTRP3 protection seen *in vivo*, Com.C (AMPK axis inhibitor) or CBO-P11 (VEGF inhibitor) was given together with rCTRP3 via the right lateral cerebral ventricle, and neurological function, BBB integrity and capillary density were assessed 7 days after ICH. As shown in Figures 7A–D, blocking AMPK activation abolished rCTRP3-induced pAMPK, HIF-1α and VEGF upregulation, and blocking VEGF activation only abolished rCTRP3-induced VEGF upregulation. In addition, CTRP3-induced capillary formation was entirely terminated when either AMPK or VEGF was inhibited (Figures 7G,H). But, the neurological protective aspect of CTRP3 was completely blocked by Com.C and partially blocked by CBO-P11 (Figures 7E,F). These results imply that, though the neurological protective effect of CTRP3 is controlled significantly by AMPK/HIF-1α/VEGF signaling-induced angiogenesis, other mechanisms also provide neurological protection to CTRP3 against ICH.

## Discussion

In the present study, we investigated the ability of CTRP3 to ameliorate secondary brain injury after ICH in rats. Based on our knowledge, this is the first report that investigated the effects of CTRP3 therapy in angiogenesis and brain injury after ICH. The main discoveries of the study are as follows: (1) exogenous rCTRP3 or Lenti-CTRP3 in ICH rats demonstrates the same tendency to attenuate ICH-induced brain injury; (2) CTRP3 promotes focal angiogenesis and attenuates ICH-induced brain edema and breakdown of the BBB; and (3) CTRP3 may exert its angiogenic effect through AMPK/HIF-1α/VEGF signaling. These findings imply that CTRP3 is a novel angiogenic factor that might perform a key part in encouraging angiogenesis by activating the AMPK signaling pathway during ICH.

The prognosis of ICH is affected by multiple factors (Brown et al., 1996). Neurotrophin family, anti-oxidative mediators, anti-mitochondrial impairment or anti-inflammatory drugs contribute to functional recovery and promote neuronal survival in the central nervous system (Ip et al., 1993; Chung et al., 2013; Xu et al., 2013; Chen et al., 2015; Wei et al., 2015). Angiogenesis induced in the ischemic penumbra (Risau, 1997) as well as rapid recovery of reperfusion and oxygen supply in injured brain tissues are critical prognostic factors (Mayer et al., 1998). In this study, we provide new evidence that CTRP3 has strong angiogenic and neuroprotective aspects, implying that CTRP3 could be an innovative therapeutic target of ICH.

It is known that compensatory angiogenesis can happen in the peri-hematoma region after ICH. Angiogenesis is a stepwise procedure that includes an increase in vascular permeability, degradation of the surrounding matrix, proliferation and migration of endothelial cells, and stabilization of freshly created microvessels (Conway et al., 2001). Concerted actions of many angiogenic molecules are necessary in this procedure, and VEGF is the most vital factor during each step of angiogenesis (Rosenstein et al., 1998; Yancopoulos et al., 2000). A number of animal experimental studies have shown that VEGF and fibroblast growth factor treatment encourages angiogenesis with ideal efficacy and increases capillary numbers (Lavu et al., 2011; Ye, 2016). However, endogenous angiogenesis following a stroke is insufficient to reverse brain injury. Our study shows that CTRP3 successfully encourages angiogenesis and upregulates VEGF expression in the striatum ipsilateral to the hemorrhage, which leads to increased vessel density. Because CTRP3 has been established to directly encourage endothelial cell proliferation and migration but not increase tube formation (a procedure that requires complexity surpassing proliferation and migration), angiogenic factors other than VEGF are likely involved.

These findings contribute to the increasing literature on the vital part of VEGF in brain injury. In fact, VEGF levels are increased during a plethora of pathological events in the brain, implicating its essential role in brain repair processes (Cristofaro and Emanueli, 2009). VEGF binds to two receptors, VEGF receptor-1 (VEGFR-1) and VEGF receptor-2 (VEGFR-2), through which it encourages revascularization and the mending of the BBB and re-establishes metabolic and trophic assistance to injured tissue (Krum et al., 2008; Shimotake et al., 2010). Future work in this field should aim to illuminate whether CTRP3 interacts with one or both receptors during ICH.

Because ICH-induced stress can change the composition, structure and distribution of the extracellular matrix, which has a vital role in creating normal brain tissue structures and is closely linked with brain injury-induced brain edema formation (Keep et al., 2012; Chung et al., 2013; Turner and Sharp, 2016), we investigated the effect of CTRP3 on BBB integrity by measuring brain water content and extravasation of Evans blue dye. We showed that angiogenesis via CTRP3 treatment resulted in preservation of the BBB. This observation in ICH extends past discoveries from other models of brain injury. In a model of cerebral ischemia, VEGF bound to VEGFR-2, which is expressed predominantly on activated astrocytes in the central nervous system, and encouraged revascularization and repair of the BBB by giving metabolic and trophic assistance to injured tissue (Krum et al., 2008; Shimotake et al., 2010; Hao et al., 2011). In a rat model of stroke, VEGF enhanced angiogenesis in the ischemic brain and reduced neurological deficits during recovery (Zhang et al., 2000). But, we were not able to demonstrate any effect of CTRP3 treatment on hematoma volume. We speculate that this may be due to use of a blood-induced ICH model.

It is important to note that the CTRP3-treated rats manifested not only greatly strengthened angiogenesis and enhanced BBB preservation but also diminished neurological deficits. There are several possibilities for this improvement. First, the great angiogenic effect of CTRP3 in the border zone may help in restoring the blood flow, thereby rescuing dying neurons around the hematoma. Second, angiogenesis may assist new neurons migrating to damaged brain regions and give trophic material to these cells (Kojima et al., 2010; Lei et al., 2013). Finally, CTRP3 may promote differentiation of neural stem cells into neurons. Such intriguing possibilities all merit express study.

Around the hematoma, ischemia and hypoxia are apparent in ICH and this stress activates the AMPK and HIF1 signaling pathway, which induces the AMPK phosphorylation in human umbilical vein endothelial cells and promotes the recruitment, migration, proliferation and differentiation of endothelial cells (Nagata et al., 2003). In ischemic mice hindlimbs, AICAR, an AMPK antagonist induces expression of endogenous HIF target gene VEGF, but dominant-negative AMPK abolishes this expression at both steady state mRNA and protein levels (Ouchi et al., 2005). These data suggest that AMPK signaling is likely to regulate the expression of VEGF and promote angiogenesis in response to ischemic injury.

CTRP3 is a paralog of adiponectin, and it is well received that adiponectin encourages angiogenesis through activation of AMPK signaling (Shimano et al., 2010). Yet, whether CTRP3 encourages angiogenesis in cerebral tissue through the same pathway is not yet known. More important, inhibiting AMPK phosphorylation by Com.C greatly eradicated CTRP3-induced HIF-1α and VEGF expression and blocked the angiogenic effect of CTRP3. These findings defend the existence of an AMPK-dependent mechanism for angiogenic effects of CTRP3.

## Conclusion

We conclude that CTRP3, a key member of a newly identified adipokine family, upregulates expression of angiogenic cytokines and induces robust angiogenesis, which led to enhanced preservation of the BBB and reduction of neurological deficits after ICH. The effect is mediated by the AMPK signaling pathway. These findings suggest that CTRP3 plays a positive role during ICH and has therapeutic potential.

## Author Contributions

SW, YaZ, YB, LL and YoZ: conceived and designed the experiments. SW, YaZ, YB and SY: conducted the experiments. SW and YaZ: analyzed the results. SW, YC and JZ: contributed materials and analysis tools. SW and YaZ: wrote the article. SW and YaZ: contributed equally to this study. All authors reviewed the manuscript.

## Funding

This work was supported by The National Natural Science Foundation of China (81271460 and 81671158), Natural Science Youth Foundation of China (No. 81301125), the Medical scientific research projects of Chongqing (20120221) and the Natural Science Foundation of Chongqing Science and Technology Committee, China (No. cstc2015jcyjA10048).

## Conflict of Interest Statement

The authors declare that the research was conducted in the absence of any commercial or financial relationships that could be construed as a potential conflict of interest.
